# Resilience in the Surgical Scheduling to Support Adaptive Scheduling System

**DOI:** 10.3390/ijerph17103511

**Published:** 2020-05-18

**Authors:** Lisa Wiyartanti, Choon Hak Lim, Myon Woong Park, Jae Kwan Kim, Gyu Hyun Kwon, Laehyun Kim

**Affiliations:** 1 Division of Nano and Information Technology, KIST School, Korea University of Science and Technology (UST), Seoul 02792, Korea; lisa.wiyartanti@ust.ac.kr; 2 Center for Bionics, Korea Institute of Science and Technology (KIST), Seoul 02792, Korea; myon@kist.re.kr (M.W.P.); kimjk@kist.re.kr (J.K.K.); 3 Department of Anesthesiology and Pain Medicine, College of Medicine, Korea University, Seoul 02841, Korea; yourejoice@korea.ac.kr; 4 Graduate School of Technology & Innovation Management, Hanyang University, Seoul 04763, Korea

**Keywords:** surgical scheduling, uncertainties, resilience, situation awareness, patient safety

## Abstract

Operating Room (OR) managers frequently encounter uncertainties related to real-time scheduling, especially on the day of surgery. It is necessary to enable earlier identification of uncertainties occurring in the perioperative environment. This study aims to propose a framework for resilient surgical scheduling by identifying uncertainty factors affecting the real-time surgical scheduling through a mixed-methods study. We collected the pre- and post-surgical scheduling data for twenty days and a one-day observation data in a top-tier general university hospital in South Korea. Data were compared and analyzed for any changes related to the dimensions of uncertainty. The observations in situ of surgical scheduling were performed to confirm our findings from the quantitative data. Analysis was divided into two phases of fundamental uncertainties categorization (conceptual, technical and personal) and uncertainties leveling for effective decision-making strategies. Pre- and post-surgical scheduling data analysis showed that unconfirmed patient medical conditions and emergency cases are the main causes of frequent same-day surgery schedule changes, with derived factors that affect the scheduling pattern (time of surgery, overtime surgery, surgical procedure changes and surgery duration). The observation revealed how the OR manager controlled the unexpected events to prevent overtime surgeries. In conclusion, integrating resilience approach to identifying uncertainties and managing event changes can minimize potential risks that may compromise the surgical personnel and patients’ safety, thereby promoting higher resilience in the current system. Furthermore, this strategy may improve coordination among personnel and increase surgical scheduling efficiency.

## 1. Introduction

Surgical scheduling has always been a challenge, especially when the operating room (OR) manager must perform same-day schedule changes. High utilization of ORs and overtime avoidance are the two most important goals of OR management in South Korea [1]. The difficulties with same-day surgical scheduling are commonly related to many factors, including possible delays or cancellations and resources availability [2,3,4,5,6]. In a recent study, causes of same-day surgery cancellations were classified as either avoidable or unavoidable and subsequently subclassified as medical, facilities or patient-related cancellations [5,6,7,8,9]. These circumstances are often unanticipated and cause uncertainties. Continuous monitoring may be a tentative solution to encounter the situation. This type of situation, hereafter referred to as uncertainty, will create immense problems if not carefully considered. For this reason, uncertainties must be taken into consideration in terms of the continuous planning of same-day surgical scheduling.

Uncertainty of surgical scheduling is considered as one of the most important factors of hospital management. Previous reports have stated that uncertainties in surgical planning and scheduling are primarily related to perioperative patients, surgery duration and resources [10,11,12,13,14,15,16]. Improper management of uncertainties can not only affect same-day surgery planning and scheduling but also increase the cost of resources [17,18,19]. Some studies proposed mathematical methods to address the emergency cases and surgery duration [20], availability of resources [20,21,22] and predicted the intervention time during a surgical procedure [23]. However, problems in daily surgical scheduling are not limited to technical problems but are also influenced by non-technical issues such as human behavior or socio-technical problems, which may not be considered by the system designer. Although a framework to model the uncertainties was developed in our previous study by identifying the dimensions of uncertainty as based on the qualitative data, improvement is necessary to account for the constantly fluctuating environment of the OR [24]. These fluctuations may be affected by medical staff movement or presence, ward overflow and surgery schedule negotiations [10,25,26,27].

In general, OR managers frequently encounter uncertainties related to real-time scheduling because they are required to make quick decisions in order to minimize risks occurring as due to unexpected events. The OR, as part of a high-reliability organization (HRO), has been extensively researched not only from the perspective of resource management but also in order to maintain a high safety climate for both patients and caregivers [28,29,30]. The concept of mindful infrastructure in HRO raises awareness of how to manage unexpected events so to escalate risks and disasters [29,30]. In research related to the HRO, the resilience model has long been known to facilitate risk and hazard identification in order to provide suitable solutions when a problem occurs. It is now also being widely applied in the field of health care services [31,32,33,34]. 

The aim of this study was to propose a framework for resilient surgical scheduling by identifying uncertainty factors affecting the real-time surgical scheduling through a mixed-methods study. This would not only investigate the technical issue but also requires attention to human factors that may affect the scheduling uncertainty. We analyzed and modified the previous framework by adopting a method of uncertainty classification as based on the sources of uncertainty and by using the Beresford model to incorporate the proposed strategy into the previous framework [24,35]. Subsequently, each group of uncertainty was categorized into a risk level, as according to the classification of risks described by Courtney, in order to provide suitable solutions as based on the level of resilience [36]. In summary, our study has been designed to accommodate the needs of OR managers who must efficiently manage both social and technical aspects related to same-day surgical scheduling strategy. 

## 2. Materials and Methods 

### 2.1. Study Design

In order to attain a systematic understanding of the real-time surgical scheduling process, we carried out a mixed-methods study, consisting of a qualitative and a quantitative phase. For the quantitative phase, we completed a data collection and analysis of surgical scheduling sheets for 20 nonconsecutive days, randomly selected days, excluding weekends and certain holidays between October and December 2015 consists of 2407 total numbers of surgical scheduling data (1016 pre-surgical and 1391 post-surgical scheduling accounts). In consideration of the uncertainties involved in surgical scheduling, we analyzed the quantitative data by categorizing each scheduling attribute that is outlined in the surgical schedule sheets and as based on the scheduling factors that have been discussed in previously reported surgical scheduling literature. 

In qualitative study, further surgical scheduling observations and interpretations were performed to confirm our findings from the quantitative data. Two researchers were tasked with observation in order to increase efficiency, as unstructured interviews were also conducted during the observation time as part of participant observation. In addition to performing data categorization, we observed activities in the ORs and used the field notes to document factual data since it was allowed during observation. The observation data were then classified as either technical or behavioral to account for both technology and human intervention in the scheduling process. Finally, we conclude our findings from these two studies. 

### 2.2. Participants and Context

Our research was conducted in a top-tier general university hospital located in Seoul, South Korea. The data were collected from both pre- and post-surgical scheduling sheets of scheduled surgeries under general anesthesia and via one-day surgery observations at the hospital site that were conducted in consideration of data from previous studies and interviews [24,37,38]. The pre- and post-surgical scheduling sheets were collected from the Department of Anesthesiology. Subsequently, observations were conducted by two researchers accompanied by the chief and a chief resident, of the Thoracic and Cardiovascular Surgery Anesthesiology Department and one anesthesiologist who is also an expert in the Critical Care Medicine. The chief resident is responsible for organizing the surgical schedule for the entire day and planning the schedule for the next day. Participants in this research except the chief resident, are the member of Korean Society of Anesthesiologist with more than ten years of surgical scheduling experience. One of them serves as the Director of the Ethics Board of the Korean Society of Critical Medicine. With extensive experience in their field, our participants have comprehensive information in the Korean surgical scheduling context.

**Figure 1:**
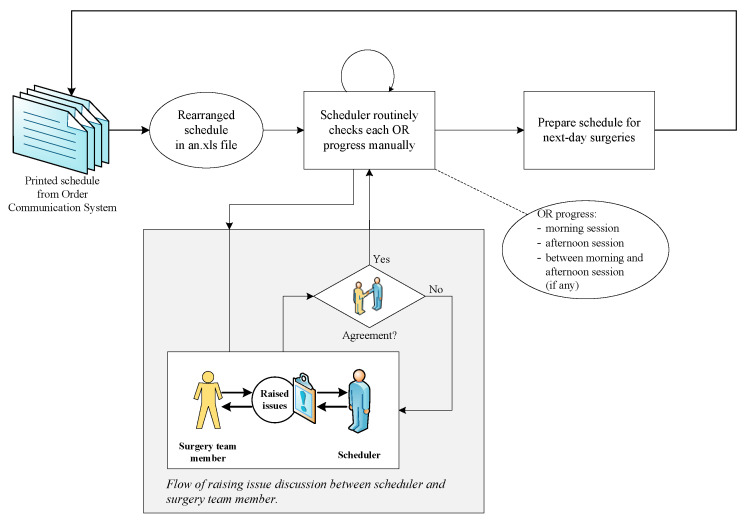
Existing surgical scheduling monitoring process.

In order to deal with complexity of the surgical scheduling, we designated the hospital in consideration of one of the largest and representative research hospitals with over 1000 beds and more than 20 operating rooms. Currently, the hospital normally operates 22 ORs for eight hours a day from 8:30 AM to 4:30 PM, with a certain number of ORs specifically allocated to each surgery department. However, because there are no ORs designated for emergency cases, OR allocation is subject to change. Among the 22 ORs, two are dedicated for robotic surgeries. The remaining 20 rooms are for general anesthesia, with two rooms being designated for local anesthesia surgeries. Additionally, one OR is only available on Tuesday, Wednesday and Thursday. The surgical scheduling is managed by the Anesthesiology Department, in which the fourth-year residents act as OR managers with senior anesthesiologists as their supervisors. Figure 1 shows the process flow diagram of current surgical schedule monitoring in our researched hospital.

In the researched hospital, each surgery department submits their daily scheduling plan via a system referred to as an Order Communication System (OCS). The OCS manages all administration processes in the surgery department, including patient information. Anesthesiologists manage the daily schedule by using the daily surgery schedule created by the OCS and received by 4:30 PM one day before a surgery. Anesthesiologists will then review patient eligibility for the procedures, check the OR availability and plan the anesthesiology arrangement. Subsequently, patient information detailing the results of all previous visits is communicated during the morning briefing on the day of surgery. The latest condition of the patient, which results from the same-day patient diagnosis and interviews, may mandate changes to the surgery schedule, thereby requiring constant updating to all surgical team members. However, this function is not supported by the OCS. 

**Figure 2:**
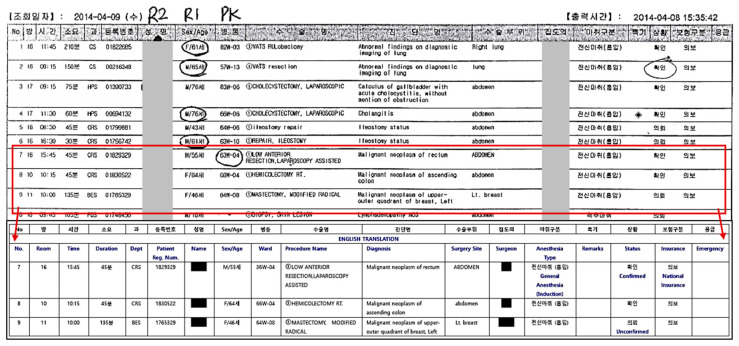
Pre-surgical scheduling list excerpted from the Order Communication System (OCS). The patient’s and surgeon’s names were masked to protect their privacy.

The schedule sheet created by the OCS is used as the primary schedule (See Figure 2). This printed schedule lists the type of surgery along with details such as OR number, the start time, duration, department, patient information, ward location, procedure, diagnosis, surgery site, surgeon, anesthesia type, confirmation of surgery and emergency status. However, up until a few hours before the surgery is originally scheduled to begin, schedule changes frequently occur in response to the patients’ condition or other unconfirmed statuses. Furthermore, although anesthesiologists continue to observe the patients during the waiting time, emergency cases may arise changes to the pre-defined schedule. In this case, the emergency surgery may occupy the time slot for an unconfirmed patient, thereby requiring that the surgery of the unconfirmed patient be postponed. 

### 2.3. Data Analysis

**Figure 3:**
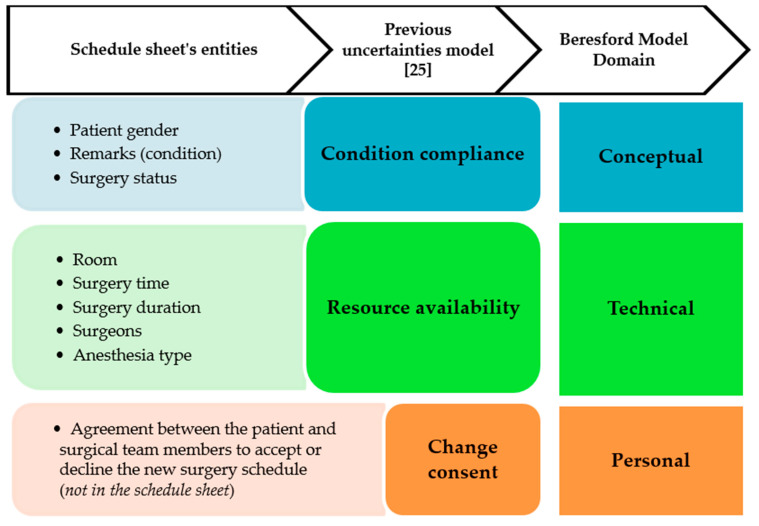
Interpretation of the schedule sheet’s parameters composed into the dimension uncertainty and clustered into Beresford Model Domain.

The schedule sheets collected from the Department of Anesthesiology were manually input into our surgical scheduling simulation database. The simulation database is used to see if there is any conflicted schedule based from the OCS. We used the descriptive statistics to summarize our schedule sheet data collection, consists of the average and standard deviation of daily surgeries, additional cases, emergency cases, surgery cancellation and surgery duration. As for the observation data, participating researchers were encouraged to take field notes. The data collected via schedule sheets and observations have been extracted as according to the uncertainties classifications and aligned as per the Beresford model of uncertainty [35]. Data from the schedule sheet have been compared and analyzed for any changes related to the dimensions of uncertainty generated in our previous study [24]. The following factors have been considered for comparison—surgery start time, OR used for surgery, emergency status and surgery status. For categorization, interpretation of significant data entities from the schedule sheet have been clustered into the Beresford model, which classifies three foundational domains of uncertainty—conceptual, technical and personal (See Figure 3). In our study, the ‘technical’ uncertainty describes the perception of current situation regarding the technical aspects of the surgery itself, ‘conceptual’ associates to the experts’ medical observation and examination and ‘personal’ uncertainty relates to the patient and caregiver’s expectation. Derivation of uncertainty factors from the schedule sheet is further explained in Section 3.1 and final cluster combined with observation result included in in Section 4.1. 

The analysis was finalized by leveling the uncertainties via the leveling model designed by Courtney for effective decision-making strategies [36]. The Courtney model mentioned the four levels of uncertainty, which were formed in consideration of the consequences of future decisions. It explains how uncertainty can be scaled into the following four levels—Level 1 (Clear-Enough Future), Level 2 (Alternate Futures), Level 3 (Range of Futures) and Level 4 (True Ambiguity). We then classified each observed factors derived from both schedule sheets and surgery observation according to relevant levels. The more detailed explanation of how we applied this leveling process is provided in Section 4.3.

## 3. Results

### 3.1. Dimension of Uncertainties Derived from the Schedule Sheet

**Figure 4:**
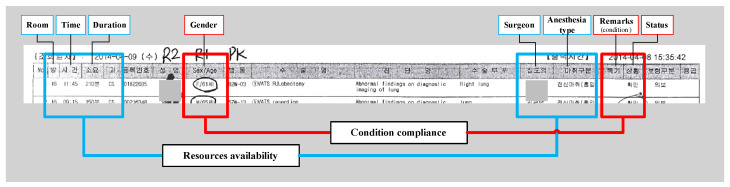
Dimension of uncertainties derived from the schedule sheet.

In our previous study, general dimensions of uncertainty were proposed as a basis for generating a mathematical model to estimate the impact value of a schedule change [24]. Based on the schedule sheet, we derived data entities that were defined as potential uncertainties. The potential uncertainties are major constraints determined by time, resources and patient condition. These data were then grouped into major data categories referred as the identified dimension of uncertainties (See Figure 4) [24]. 

General dimensions were derived from the schedule sheet include condition compliance and resource availability, while change consent came from the legal factor of any schedule changes that occur. ‘Condition compliance’ defines the latest report on patient condition and is the highest priority consideration for the scheduling decisions. ‘Resource availability’ identifies the existence of resources such as ORs, surgical devices and medical staffs (i.e., anesthesiologists, surgeons and nurses). ‘Change consent’ refers to the agreement between the patient and surgical team members to accept or decline the new surgery schedule. Factors considered related to condition compliance are patient gender, special tag of surgery and surgery status. Factors considered related to resources availability are room, surgery time, surgery duration, surgeons and anesthesia type.

### 3.2. Prevalence of Surgeries

**Figure 5:**
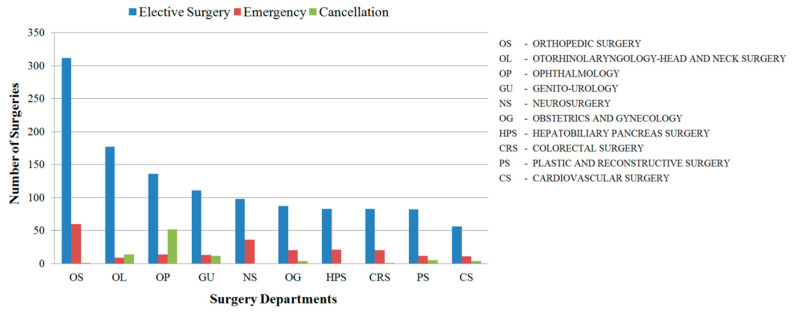
Surgery departments with highest surgery rates according to post-surgery scheduling data in this hospital.

As has been mentioned, the pre-surgical schedule sheet was prepared and printed by 4:30 PM one day before the surgery. This data yields information on the different statuses of elective surgeries; these statuses are defined as confirmed, unconfirmed, or reserved, according to the patient readiness and OR availability. Thus, schedule changes are likely to occur up until first surgeries start at 8:30 AM the next day. 1016 pre-surgical and 1391 post-surgical scheduling accounts were collected as data. In this hospital, the Orthopedic Department has the highest number of surgeries, followed by the Otorhinolaryngology-Head and Neck Department and the Ophthalmology Department. The surgical scheduling data summary is presented in Figure 5 and Table 1. 

The Orthopedic Surgery (OS) Department has the highest number of elective and emergency surgeries (See Figure 5). For elective surgeries, this may be because of the relatively short surgery duration, the low procedural complexity and few required surgical devices. Consequently, this allows the OS Department to perform many surgeries and use any OR available on the scheduled day of surgery. This assumption was confirmed via the field observation results, which are detailed in the next subsection and shown in Table 2. Conversely, some surgeries may require long durations. The longest surgery lasted for 750 min in OR 9. It was performed by a surgeon from the Otorhinolaryngology-Head and Neck Surgery (OL) Department. The OL Department is one of a few departments that perform robotic-device surgery; other departments include the Genito-Urology (GU) and Colorectal Surgery (CRS) Departments. The surgery durations for these three departments, in addition to the Thoracic & Cardiovascular Surgery (CS) Department, are significantly longer than those for the remaining departments.

### 3.3. Pre- and Post-Surgical Scheduling Differences

The result of the Shapiro-Wilk test performed on the data of daily additional surgery cases indicated that the data are normally distributed (*p*-value = 0.227 > 0.05). The post-surgical volume was significantly higher based on the Paired Sample T-Test (*p*-value = 2.2124 × 10^−10^ < 0.05), accounted for ±43% of average daily additional cases (See Table 1). The occurrence of overtime surgeries was also found to be significantly different between the pre- and post-scheduling arrangements. Here, we defined the ‘surgeries possibly out of office hours’ as all surgeries that may start or finish outside the normal office hours (08:00–16:30). Thus, a portion of these surgeries occurred during office hours. Conversely, the ‘surgeries not during office hours’ assignment refers to the surgeries with no time period overlapping with normal office hours. Additionally, emergency surgeries comprised a significant percentage of daily surgeries (18%) for the 20 days of collected data. Similarly, ‘additional cases,’ which refers to surgeries not on the previous day schedule, were also found to occur with high frequency. For a more comprehensive analysis, we conducted a field observation to validate the assumptions formed as based on the schedule data collection results.

Regarding our analysis on uncertainties, the data allowed us to form preliminary assumptions regarding unexpected event triggers. Furthermore, we derived some factors that affect the scheduling in this hospital as based on the data presented in Table 1. These factors are as follows—“(1) time of surgery, (2) overtime surgery occurrence, (3) surgery changes and (4) surgery duration. In our study, the time of surgery was blocked and categorized into three sessions—morning, mid-daytime and afternoon sessions.

### 3.4. Observation Results

The observation data was collected by two researchers. When the morning observation began, all rooms were prepared for the first surgery of the day. Two anesthesiologists accompanied the researchers during observations. An anesthesiologist (P1) introduced all ORs available in the hospital, explained the current basic schedule running strategy and introduced the chief resident in the Department of Anesthesiology who arranged the one-day schedule. The chief resident (P2), who is also the OR manager, carried the printed surgery schedule (Figure 2) and another sheet of paper that showed operating rooms map with provided details on the anesthesiologists in-charge, surgery departments and surgeon lists. Even though all schedules are planned through OCS, OR manager uses the printed schedule for practicality to manage the schedule updated in real-time. On the day of surgery, room assignment is divided into morning and afternoon sessions, which are determined every day by 8:30 AM and between 10:30 AM and 12:00 PM, respectively. In Table 2, a list of surgery departments active on the day of observation is presented. 

During observation of the morning session, the researcher was briefly allowed to enter the ORs in which P1 and P2 administered the anesthesia. The field notes comprised direct explanations from P1 and P2 about the basic surgical scheduling routines and example of cases that required schedule change, observations on surgical team behavior and short conversations between surgeons and OR managers. These findings were then categorized as based on relevance to one of the following factors of surgical scheduling—preparation, indeterminate schedule, communication, schedule updates and scheduling conflicts. Table A1 reports the findings and their categorization.

#### 3.4.1. Preparation

‘Preparation’ is a process of organizing events or activities that should be done prior to performing the surgical procedures. Before the morning session began, the OR manager assigned the rooms for 80 elective surgeries. Note that, on the observation day, many surgeries were scheduled ahead of an upcoming national election and conferences that should be attended by most of the medical doctors. Consequently, on that day, all surgeries should not be delayed to other days. The OR manager, who was an administered anesthesiologist in OR 16 on observation day, then listed all the attending surgeons and anesthesiologists. Confirming all first surgeries is a compulsory procedure to ensure that the patients, surgical team members, ORs and required medical devices are prepared for surgery as usual. Comprehensive preparation enabled all first surgeries to begin on time at 8:30 AM.

#### 3.4.2. Indeterminate Schedule

‘Indeterminate schedule’ refers to plan or unplanned schedules that could not be finalized due to unforeseen circumstances such as sudden occurrence of emergency cases. With an atypically large number of surgeries on the observation day, the OR manager was unsure as to whether all scheduled surgeries could be completed. This also affected the attending surgeons, particularly those scheduled to perform an afternoon session surgery. The absence of an automated schedule monitoring system requires that the OR manager continuously manually update the schedule by circulating each OR. Thus, the OR manager must carefully plan and frequently check the current occupancy status of each OR.

#### 3.4.3. Communication

‘Communication’ is a term related to the activities in exchanging information among the stakeholders with various communication devices. Excluding face-to-face communication, all information is updated via a short message service (SMS), an instant messaging service (IM) or telephone for more effective communication. The OR manager has two cellular phones that he carries throughout the day. One is for personal use, whereas the other cellular phone is the department cellular phone, which he uses to communicate the room assignment or information updates to other departments during a surgery. (Note that they are solely used for surgery communication purposes.) Likewise, every department has one cell phone for dedicated use as a form of communication to others within the ORs.

#### 3.4.4. Schedule Updates

‘Schedule updates’ is an action to integrate and convert the up-to-date information to the planned schedules. An anesthesiologist who is tasked with the OR manager role receives demands for continuous and immediate schedule updates from other surgery team members. Thus, being an OR manager needs to be proactive with respect to maintaining surgery progress updates for each OR. During this time, the basic check and re-check procedures were still manually performed by visiting each room to confirm the up-to-date surgery progress or via cellular phone communication. During the morning and afternoon sessions of observation, the OR manager was still undecided on whether to cancel or perform the remaining elective surgeries in the evening.

#### 3.4.5. Scheduling Conflict

‘Scheduling conflict’ defines the incompatibility or overlapping related to the surgical scheduling process. Conflicts related to OR scheduling often arise because surgeons must complete a target number of surgeries in a day, while the anesthesiologists must make sure that the patient is in a stable and acceptable condition for each surgery. When many surgeries are scheduled for a single day, there needs to be mutual understanding and good cooperation between all surgical team members to avoid cancellations. Because high pressure in the surgical environment is an unavoidable condition, the risks of human or technical error must be minimized. This is also necessary to maintain the highest standard of patient safety practices.

## 4. Discussion

### 4.1. Categorization of Uncertainties

Uncertainties commonly arise, making daily schedule maintenance a challenge for OR managers. Thus, to design a more accurate uncertainty model, we have specified dimensions that have been identified via the pre- and post-surgical scheduling data comparison and surgery observation; consequently, we have updated our dimensions of uncertainty as outlined in Table 3. We grouped our findings as based on the three characteristics of the Beresford model of uncertainty. The model has been successfully applied and extended to improve uncertainty-based decision making and performance measurement efficacy in the medical field [39,40,41,42].

By considering the categorized dimensions listed in Table 3, we identified there are many dynamic situations can increase the scheduling complexity. One crucial consideration is how to raise awareness among the surgical team members and OR managers in order to facilitate the scheduling process; this is related to communication, as has been demonstrated via Scene 4. OR managers undertake dual tasks. The first task is to expect frequent updates that can be easily accessed and monitored at any time and from any location in the hospital. In addition, they should perform the task of a general anesthesiologist. Since an awareness system is able to support their needs has not yet been developed, the OR managers send and receive messages via traditional communication channels or social network messaging services to stay informed. When necessary, OR managers may also take time to visit each room for a short duration to monitor the surgery progress. However, this event may yield an unexpected and/or undesirable outcome [43]. Thus, a better strategy is needed such that OR managers have constant access to the most up-to-date information. 

Another dynamic problem brought to our attention following the interview with the OR manager was related to the surgery duration. In the current scheduling system, the OR manager cannot precisely plan the elective surgeries schedule because the surgery duration may not be accurately reported by the surgery department. As an example, although a surgery is scheduled to finish within 30 min, more time is actually required; this causes the attending surgeons to request additional time for surgery (Table A1: Scene 7). Because such events are common, the data in the scheduling system should be sufficiently reliable to be used as the basis for schedule planning [13,14,15,44]. Moreover, when a situation occurs, the OR managers must be able to determine which actions should be taken to avoid a delay or to minimize the consequences.

### 4.2. Resilience for Scheduling Change Management

Resilience has been implemented as a guideline protocol for uncertainty management because it has been proven to be a good approach to manage uncertainties in the HRO and particularly in the OR [31,45]. In terms of our research on surgical scheduling, resilience can help the OR managers better balance their dual responsibilities and make more efficient strategic decisions for real-time scheduling. OR manager has also established some decision-making attitudes toward the “principles of anticipation” and the “principles of containment,” which are the two mandatory principles required to establish resilience [29,46]. In order to achieve resilience in surgical scheduling, both of these principles were applied to our framework.

**Figure 6:**
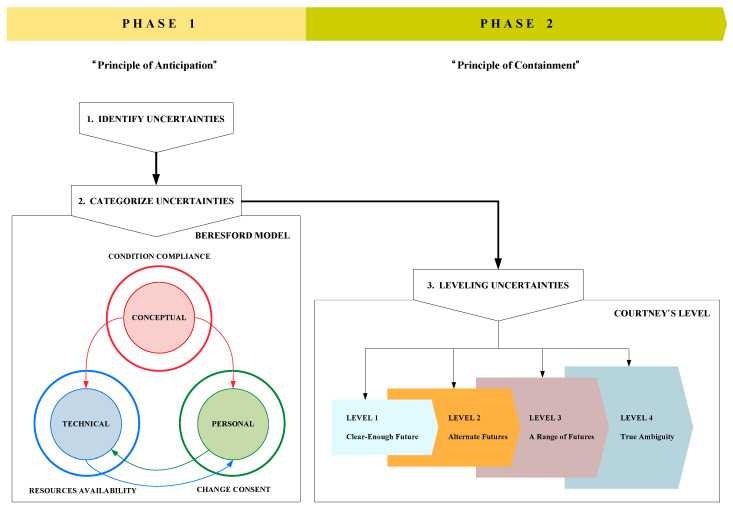
Proposed framework; the Beresford model of uncertainty and Courtney uncertainty level concept has been integrated to achieve higher resilience in a mindful infrastructure.

Our framework consists of two phases. The first phase entails identification and categorization of uncertainties via the Beresford Model. The second phase requires that these categorized uncertainties be projected onto the leveling model developed by Courtney et al. [47]. This method can help OR managers better manage uncertainties and unscheduled events and determine which strategy and solution will most effectively minimize consequences. Thus, via this method, continuous planning is optimized for surgical scheduling. An overview of the proposed procedural assessment to achieve resilience in a mindful infrastructure is depicted in Figure 6. 

An uncertainty is either predictable or unpredictable in conventional management practices. However, in reality, events can be unpredictable with different levels of uncertainty and require a wide variety of future strategic decisions. We employed the Beresford model as our approach to categorize uncertainties. It simplifies the process of identifying relevant solutions when problems arise. However, in order to build a resilient system that can efficiently manage uncertainty and dynamic problems, an additional approach is required to predict possible outcomes as based on the strategies and solutions that can be recommended. The uncertainty leveling model is considerably more precise than the conventional management practice. Hence, to more efficiently manage the uncertainties and dynamic problems that are specific to the scheduling of surgeries, we have integrated the Beresford model and procedural assessment proposed by Courtney into our dimensions model. This strategy can be implemented as the foundation for developing uncertainty anticipation rules. 

Various examples applied Courtney model were presented in the succeeding research report on making decisions for uncertainty assessment [47,48,49]. Although the model was initially designed to address problems common to business and marketing, the concept itself can actually be applied to any industry prone to uncertainties. Thus it incorporates a strategic decision planning model. In our case, the framework has been customized to determine strategies and solutions for problems related to ORs, because the framework can visualize details not only related to safety risks but also to the economic consequences. 

### 4.3. Applications of the Proposed Framework

Applying the concept of uncertainty to our framework generates the classification. The change of patient condition can apply to all level uncertainties. According to the American Society of Anesthesiologists (ASA), the physical status classification system categorizes six groups of patient conditions ranging from “a normal healthy patient” to “a brain-dead patient” who requires further procedures [50]. For example, in Level 1 uncertainty, schedule cancellation is mostly caused by the inaccurate assessment of patient medical condition. This cancellation may require a change from a normal elective surgery to an emergency case. The emergency cases are classified as a Level 1 uncertainty because the decision that resulted in urgent and immediate action is clear. However, this emergency surgery may cause other events. Other surgeries can be delayed or canceled because of the unavailability of ORs, thereby amplifying the potential effects of the event such that it may require classification as either Level 2 or Level 3. Additionally, if hospitals do not have dedicated ORs for emergency cases, OR managers must think of optimal and feasible solutions, to minimize consequences for all involved stakeholders. 

Similarly, as for the “patient care” consent, no action would be taken without family consent unless the situation is an emergency, is classified as a case of Level 1 uncertainty. Examples of Level 2 uncertainty classification are related to OR availability and surgery duration. The effects of an unavailable OR may engender different consequences, such as schedule changes or overtime. This event is related to the availability of resources such as anesthesiologists, nurses or medical devices. Prolonged surgery duration may cause delay or cancellation of other surgeries.

**Figure 7:**
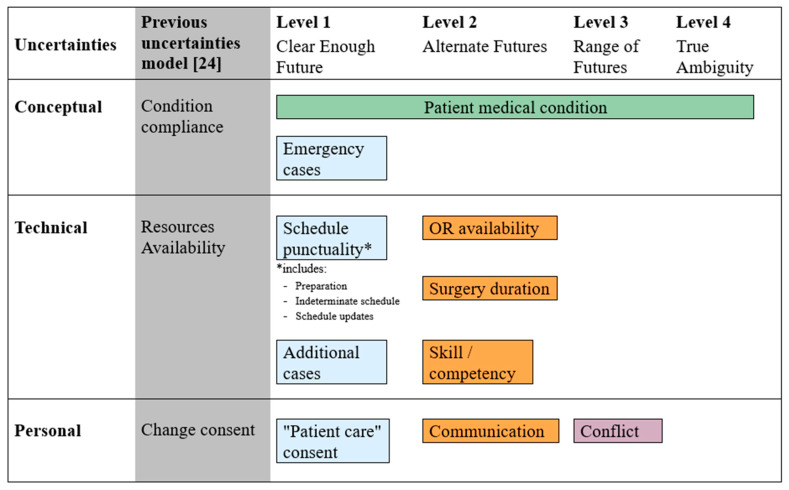
Leveling the identified and categorized uncertainties as per the Courtney level of uncertainty model.

Furthermore, the situation can be complicated whenever there were additional procedures with ill-prepared equipment. In the case of a Level 3 or 4 uncertainty, we may include major accidents or natural disasters in these categories. Although we believe that the hospitals generally have a national standard operating procedure that is able to handle large numbers of casualties from unexpected accidents or unanticipated natural disasters, these probable catastrophes must be taken into consideration. Figure 7 shows the complete leveling scheme for the identified uncertainties. 

In this study, some examples are presented on how principles of the mindful infrastructure is being applied in this particular surgery department despite the lack of system support for the OR managers. The OR manager has demonstrated how confirming the first surgery of the day may prevent the delay of the first surgery from creating delays of subsequent surgeries (Table A1: Scene 1); consequently, because of the due diligence of the OR manager, this situation remained as a Level 1 uncertainty. Alternatively, when the OR manager decided to cancel two surgeries because of the unstable medical condition of the patients despite the insistence of the attending surgeons to proceed (Table A1: Scene 7), the OR manager has changed the level of uncertainty from Level 1 to Level 2. This decision was made as based on previous work experiences because the consequences resulting from possible overtime surgery (i.e., patient safety, reduced surgeon concentration that would lead to human errors) would be more significant than canceling the surgeries. 

After reviewing the entire process of categorization and leveling the uncertainties, we have concluded that in real-time scheduling progress must provide relevant information to facilitate the identification and categorization of uncertainties, as well as to improve situation awareness.

### 4.4. Toward an Adaptive Scheduling System

Studies have shown that building a good communication infrastructure in clinical environments is essential. Especially when the coordination among surgery department personnel is critical, the communication breakdown in the perioperative surgery environment may lead to serious problems [43,51,52]. Issues of personnel view, capability and awareness also have been addressed to improve resilience of the system, as necessary as adaptation to work expectation in the working environment [53,54,55,56,57,58]. In this study, the OR manager has demonstrated that they are capable of managing both roles as an anesthesiologist and OR manager by maintaining communication with other surgeons and anesthesiologists (Table A1: Scene 2, 3, 4, 5). However, the observations and interviews also helped us to understand that the work of OR managers to manage manual schedule updates with the limited support from information technology. They also have to focus and concentrate on the task of intrinsic anesthesiologist. Such dual duties are highly strenuous. Thus, it is necessary to develop a system to support OR managers by facilitating awareness of all schedules changes, irrespective of time and place. 

A system that records steps involved in a surgical procedure can predict more accurate surgery time, thus improve the calculation of surgery duration for similar procedures [59,60]. Another system for total resource management to control OR resource availability, such as surgical teams and medical devices, is also crucial especially if it can be integrated into the real-time scheduling information system [61]. Our purposed framework for uncertainties provides theoretical foundations for an adaptive scheduling system.

Situation awareness among the surgical team members and OR managers was the primary focus of our previous work [37]. There are three levels of situation awareness, which are defined as follows—(1) perception of events in the surrounding environment, (2) comprehension of the events and current situation and (3) prediction of the future status of the events [62]. Therefore, it can be ascertained that the proposed framework can be implemented in conjunction with the concept of situation awareness to improve scheduling efficiency in a surgical environment. 

In order to improve scheduling accuracy, various surgeries can be periodically observed to determine the duration and how emergency cases may affect the current schedule can be analyzed [63,64]. OR managers must efficiently manage both social and technical aspects related to same-day surgical scheduling strategy. Considering the aim of our study, it can be viewed as being closely related to the socio-technical system design that has been described in previous research [65,66,67]. As observed, the issues of authority in the organization also emerged. Surgeons often assigned surgeries as emergency in order to complete as many surgeries as possible within a day, although, this issue was not our focus in this study. Therefore, considering coordination, communication and hierarchical constraints, the system designer should more thoroughly consider the socio-technical system values. In order to manage the system changes, behavior-based problems are occasionally more significant than technical-based problems [67,68]. 

## 5. Conclusions

Uncertainties or unexpected events inevitably occur in an HRO such as a hospital, especially the surgery department and OR manager behavior was considered as one of factors that determine the success of surgical scheduling management system. We identified uncertainties factors that affected real-time surgical scheduling by analyzing schedule sheets and observing the OR activities. Thus, this study attempted to develop a framework for resilient surgical scheduling that manages uncertainties that may arise on the day of a surgery. The OR managers in the hospital researched in this study are also anesthesiologists who perform dual roles. Under these circumstances, it is critical that OR managers maintain a considerably high level of focus on their tasks as an anesthesiologist, in addition to schedule arrangement. Therefore, finding resilience framework-based suitable solutions, as have been proposed in this study, is required when uncertainties that affect scheduling are present. If we know how to identify and categorize those events, then we can determine strategies to anticipate potential hazards during perioperative periods. Hence, the OR manager will be able to de-escalate and minimize undesirable consequences that may affect the safety of both surgical personnel and patients. In addition, although the OR managers were effective in preventing overtime surgeries, it was evident that managing multiple roles is not an easy task; this is primarily because the necessity of focusing and working on specific duties under time constraints and with minimum error, requires substantial effort. 

The findings from this study provided insight into how OR managers manage an immense number of surgeries. However, if we can get more perspectives from other stakeholders regarding the scheduling process and observations, then it would be advantageous to enhance our understanding on scheduling efficiency and continuous planning. In case of the scheduling data, it would be more beneficial if actual surgery times for every procedural step are acquirable. This would help predicting the surgery durations and reducing overtimes by maximizing the surgical time slots available. In order to generalize our theory, we also need input from more hospitals in other countries, since our data is limited only to the perspective from hospital in South Korea. As part of our future work, incorporating the proposed framework into the current day scheduling system is necessary. Following the framework integration, evaluation of uncertainties can be done using quantitative approach in order to calculate the risks and intangible benefits in a greater detail.

## Figures and Tables

**Table 1:** Summary of 20 days pre- and post-surgical scheduling data collection.

Measurement	Pre-Surgical	Post-Surgical
**Data Collection**		
Data collection start date	26 October 2015	26 October 2015
Data collection end date	4 December 2015	4 December 2015
Number of days	20 days	20 days
Number of surgeries	1016	1391
Average surgeries per day	50.80	69.55
Standard Deviation	7.46	13.14
**Time of Surgeries**		
Morning Session (08:30–12:00)	613	808
Mid-Daytime Session (12:00–13:00)	157	222
Afternoon Session (13:00–16:30)	246	361
**Overtime Surgeries**		
Surgeries during office hours *	933	1305
Surgeries possibly out of office hours	83	86
Surgeries not during office hours	32	45
**Surgery Changes**		
Surgery schedule changes		141
Operating room changes		326
Additional cases (average **/std. deviation)		437 (21.85/8.07)
Emergency cases (average **/std. deviation)	10	268 (13.40/4.21)
Cancellation (average **/std. deviation)		63 (3.15/1.93)
**Surgery Duration**		
Maximum surgery duration (minutes)	750	750
Minimum surgery duration (minutes)	12	12
Average/std. deviation (minutes)	79.44 (67.95)	80.78 (69.38)

* Office hours: 08:30:00–16:30:00; ** per day.

**Table 2:** Surgery departments attended the observed day of surgery.

Surgery Department	Operating Room
Otorhinolaryngology-Head and Neck Surgery	OR 1(R *), OR 9
Neurosurgery	OR 2, OR 3
Orthopedic Surgery	OR 4, OR 19, OR 20
Plastic and Reconstructive Surgery	OR 5, OR 13
Thoracic & Cardiovascular Surgery	OR 7, OR 16
Genito-Urology	OR 8
Transplantation Vascular Surgery/Colorectal Surgery	OR 10
Breast and Endocrine Surgery	OR 11
Ophthalmology	OR 14
Obstetrics and Gynecology	OR 15
Hepatobiliary Pancreas Surgery	OR 17
Transplantation Vascular Surgery	OR 18

* Robotics surgery.

**Table 3:** Dimensions of uncertainty according to the Beresford model domain.

Beresford Model Domain	Previous Uncertainties Model [24]	Observed Factors		Evidence (*Scenes are from Table A1*)
		(From Schedule Sheets)	(From Observation)	
Conceptual uncertainty	Condition compliance		*Patient medical condition* ^1^	Scene 3, Scene 8
		Emergency cases		Table 1
				
Technical uncertainty	Resource availability	Schedule punctuality	Preparation, Indeterminate schedule	Scene 1, Scene 2, Scene 6, Scene 7
		OR availability	Communication, schedule updates	Scene 2, Scene 3, Scene 5
			*Surgery duration, skill/competency* ^1^	Scene 2, Scene 7
		Additional cases		Table 1
				
Personal uncertainty	Change consent		*“Patient care” consent* ^1^	Scene 8
			Communication	Scene 3, Scene 4
			Scheduling conflict	Scene 7, Scene 8

^1^ Observed factors related to patients and surgeons.

## Abbreviations

The following abbreviations are used in this manuscript:

OR	Operating Room
HRO	High-Reliability Organization
OCS	Order Communication System
OS	Orthopedic Surgery
OL	Otorhinolaryngology-Head and Neck Surgery
GU	Genito-Urology
CRS	Colorectal Surgery
CS	Thoracic & Cardiovascular Surgery
SMS	Short Message Service
IM	Instant Messaging Service
ASA	American Society of Anesthesiologists

## Appendix A

**Table A1:** Observation category based on the observed results.

Observation Category	Observed Results
Preparation	*Scene 1*: At approximately 8:15 AM, the OR manager began visiting each OR, confirmed its first surgery and instructed the attending surgeon to proceed on time at 8:30 AM. After all first surgeries had been confirmed, the OR manager returned to his appointed surgery by 8:30 AM and prepared anesthesia induction to his patient in OR 16.
Indeterminate schedule	*Scene 2*: At 2:20 PM, Surgeon S1 asked the OR manager whether it was possible to perform his scheduled surgery at 3:00 PM in OR 18. However, at this time, the OR manager could not confidently answer, because the current ongoing surgery started late and was still in progress; thus, he asked Surgeon S1 to wait and see if this surgery could finish by the scheduled time.
Communication	*Scene 3*: The OR manager received a phone call from Surgeon S1 at 3:00 PM asking whether he could use OR 18 for a transplantation vascular surgery. The OR manager then asked Surgeon S1 whether the surgery in progress was near conclusion and the patient of Surgeon S1 was ready for the surgery. Surgeon S1 replied that the current surgery was almost finished and his patient was ready for the surgery. The OR manager confirmed to Surgeon S1 that he could prepare the surgery in OR 18. Note that the OR manager and Surgeon S1 were in different rooms.
	*Scene 4*: At 11:15 AM, Anesthesiologist P3 visited OR 16 and confirmed to the OR manager that the next surgery at 1:00 PM in OR 6 could proceed as scheduled. She explained the most up-to-date medical status of the patient and assured the OR manager that the patient was ready for the surgery. The OR manager updated the afternoon session and continued the room assignment for the afternoon session.
Schedule updates	*Scene 5*: The OR manager checked the in-progress schedule for each room whenever he was not occupied by the surgery in OR 16. During a schedule inspection, the OR manager found that the current surgery was near completion; thus, he immediately contacted the attending surgeon for the next surgery and told them to prepare.
	*Scene 6*: The OR manager received a phone call from Anesthesiologist P4 at 4:20 PM and was informed that the last surgery in OR 6 had been completed. The OR manager then asked Anesthesiologist P4 to close the OR and he updated the room assignment on his paper by marking an “X” on OR 6 the note that the room is closed for the day.
Scheduling conflict	*Scene 7*: When the OR manager visited each OR before the morning session, Surgeon S2 approached him to extend the OR time. Surgeon S2 requested additional time, stated reasons for the request and argued that it was necessary. However, the OR manager declined his request because of the vast number of surgeries that day. He asked Surgeon S2 to manage their time and proceed with the surgery as according to the reserved schedule.
	*Scene 8*: At 4:40 PM, the OR manager received a phone call from Anesthesiologist P5, who informed him that the remaining two surgeries could not be performed that day. Anesthesiologist P5 explained that the unstable condition of each of the patients proved unfavorable conditions for surgery but the surgeons continued to insist on performing the surgery. Consequently, the OR manager confirmed to Anesthesiologist P5 that the two surgeries in question would be postponed until the next day. This means that the OR manager canceled the surgeries despite the insistence of the attending surgeon.
